# New insight into anomalies of coronary arteries in children: diagnosis, treatment with perioperative and postoperative course and prognosis

**DOI:** 10.1186/s13052-026-02302-w

**Published:** 2026-07-10

**Authors:** Kamila Kurkiewicz-Sawczak, Filip Baszkowski, Filip Sawczak, Malwina Baturo, Kinga Walska-Świerc, Rafał Surmacz, Dominika Wysocka, Marta Kałużna-Oleksy, Waldemar Bobkowski

**Affiliations:** 1 Department of Paediatric Cardiology, Poznan University of Medical Sciences, Szpitalna 27/33, Poznan, 60-572 Poland; 2 1st Department of Cardiology, Poznan University of Medical Sciences, Dluga 1/2, Poznan, 61-848 Poland; 3 The Student Scientific Society of Poznan University of Medical Sciences, Poznan, Poland

**Keywords:** Congenital heart defect, Coronary artery anomalies, Heart failure with reduced ejection fraction, Ejection fraction, Paediatric cardiology

## Abstract

**Background:**

Coronary artery anomalies (CAAs) are rare and heterogeneous congenital abnormalities. However, some of them are associated with serious consequences, including myocardial ischemia, heart failure, and even death. This study aimed to characterize symptoms, course, treatment, and prognosis of different types of CAAs.

**Methods:**

This single-centre retrospective cohort study included 38 consecutive paediatric patients hospitalised in the Department of Paediatric Cardiology due to a diagnosis of CAAs between 01.2012–03.2025. Patients were divided into two groups: anomalous origin of the coronary arteries (Origin anomalies) and coronary artery fistulae (CAF), respectively, 20 and 18 patients. The collected data included clinical symptoms, laboratory tests, electrocardiography, echocardiographic parameters, course of treatment and long-term follow-up.

**Results:**

The mean age of the analysed group was 3.32 ± 5.35 years (range: 1 day − 16 years; IQR0-4.25). The study group included 42% males. During the follow-up, no patient died. There were significant differences between groups in the occurrence of dyspnoea (*p* < 0.001), cyanosis (*p* = 0.048), echocardiographic parameters: mitral insufficiency (*p* = 0.001), left ventricle dilatation (*p* < 0.001), left ventricular end diastolic diameter z-score (*p* = 0.023) and end systolic diameter z-score (*p* = 0.004) and lower ejection fraction (*p* = 0.001), which was more prevalent in the origin anomalies group than in the CAF group. Moreover, lower haemoglobin (*p* = 0.006) and haematocrit (*p* = 0.005) were observed in the origin anomalies group compared to the CAF group.

**Conclusions:**

Our study showed that prompt diagnosis and treatment, including transcatheter or surgical intervention, were associated with clinical improvement and no mortality observed during the follow-up period in the study group. Symptoms like dyspnoea, cyanosis, syncope, no weight gain, feeding disorder and fatigue, especially in younger children, should prompt us to expand the diagnosis and look for diseases such as CAAs. Additionally, patients with origin anomalies had more frequent cyanosis, dyspnoea, mitral insufficiency, left ventricle dilatation and lower ejection fraction, haematocrit, and haemoglobin levels than patients with CAF.

## Graphical abstract

**Figure:**
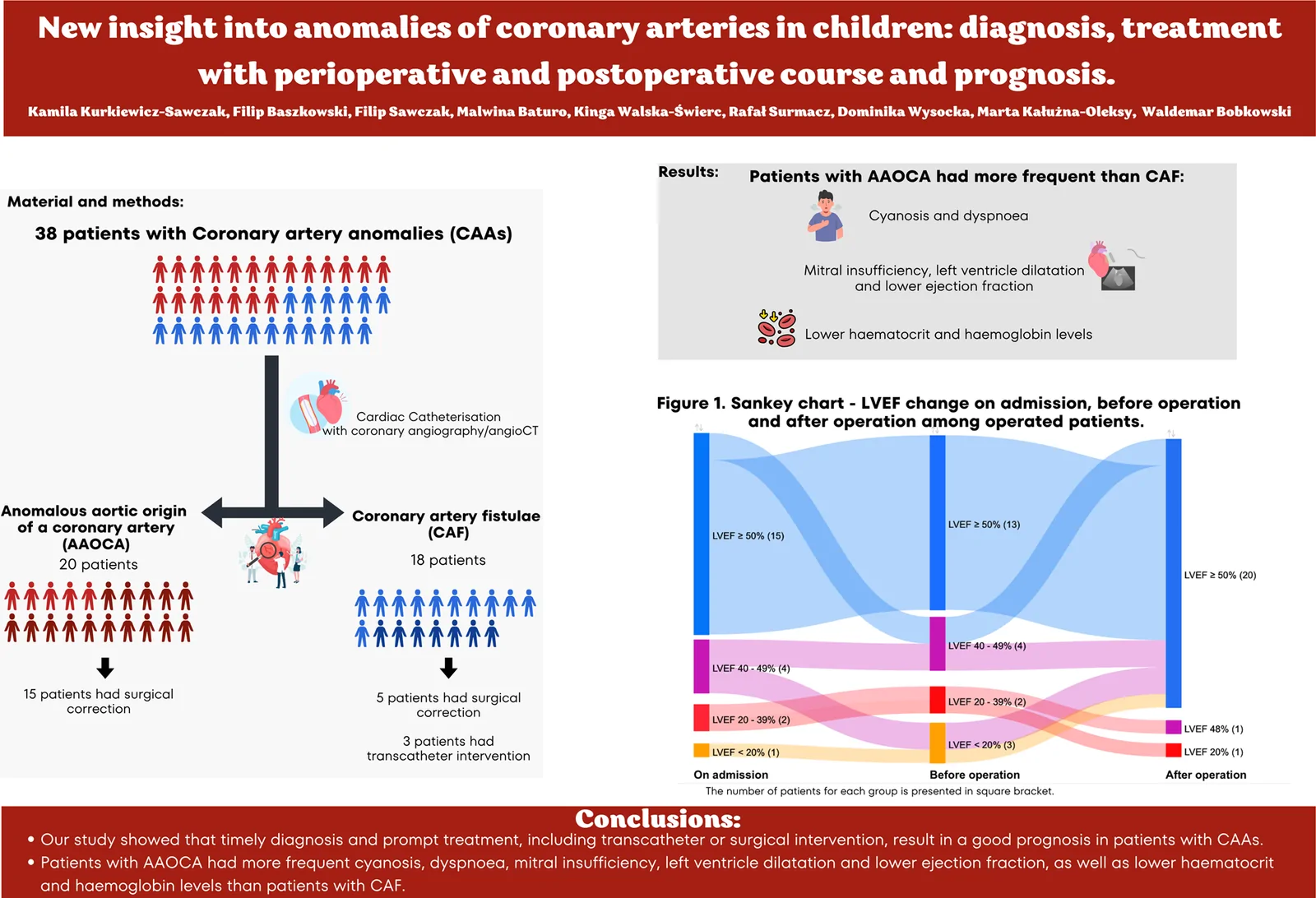

## Introduction

Coronary artery anomalies (CAAs) in children represent a heterogeneous group of structural abnormalities of the coronary arteries, present at birth and characterised by variable anatomical presentations, clinical manifestations, and prognostic implications. Although rare, they pose a significant diagnostic and therapeutic challenge in paediatric cardiology [1]. The estimated prevalence in the general population ranges from 0.21% to 5.79%, depending on the imaging modalities employed and the diagnostic criteria applied [2]. However, data regarding the prevalence of CAAs specifically in the paediatric population remains limited.

A standardised classification system and a comprehensive understanding of the aetiology of these anomalies are still lacking [3]. These congenital abnormalities affect the structure, the location, the course or the origin of the coronary arteries.

Currently, there is no universally accepted classification or international guidelines for CAAs. The literature describes multiple classifications based on angiographic patterns or a risk stratification [3, 4].

Due to the complexity and ambiguity of the classification systems, a simplified approach proposed by Gentile et al., including origin anomalies, anomalies of course, and anomaly of termination (with coronary arteriovenous fistulas and coronary stenosis) has been used [5]. Our population included anomalous origin of the coronary arteries (origin anomalies) and coronary artery fistula (CAF). No anomalies of course and coronary stenosis were reported in our population.

The clinical significance of CAAs is highly dependent on the specific anatomical configuration. Clinical manifestations as well as the prognosis are diverse. While some patients remain asymptomatic, others may present with symptoms of myocardial ischemia, heart failure (HF), such as exertional dyspnoea, syncope, or even sudden cardiac arrest [1, 3].

Diagnosis of CAAs relies primarily on imaging techniques such as transthoracic echocardiography, computed tomography angiography, coronary angiography, and cardiac magnetic resonance imaging [3, 4]. Failure to detect these abnormalities early significantly increases the risk of complications, especially in children and adolescents.

Despite the advancements in diagnostic and therapeutic modalities, further research is needed to elucidate the aetiology, natural progression, and optimal management strategies for CAAs in children. We analysed patients under 18 years of age, diagnosed with CAAs. This study aims to describe the clinical presentation, treatment, perioperative course, and follow-up outcomes in a retrospective cohort of paediatric patients with CAAs.

## Methods

### Participants

**Fig. 1:**
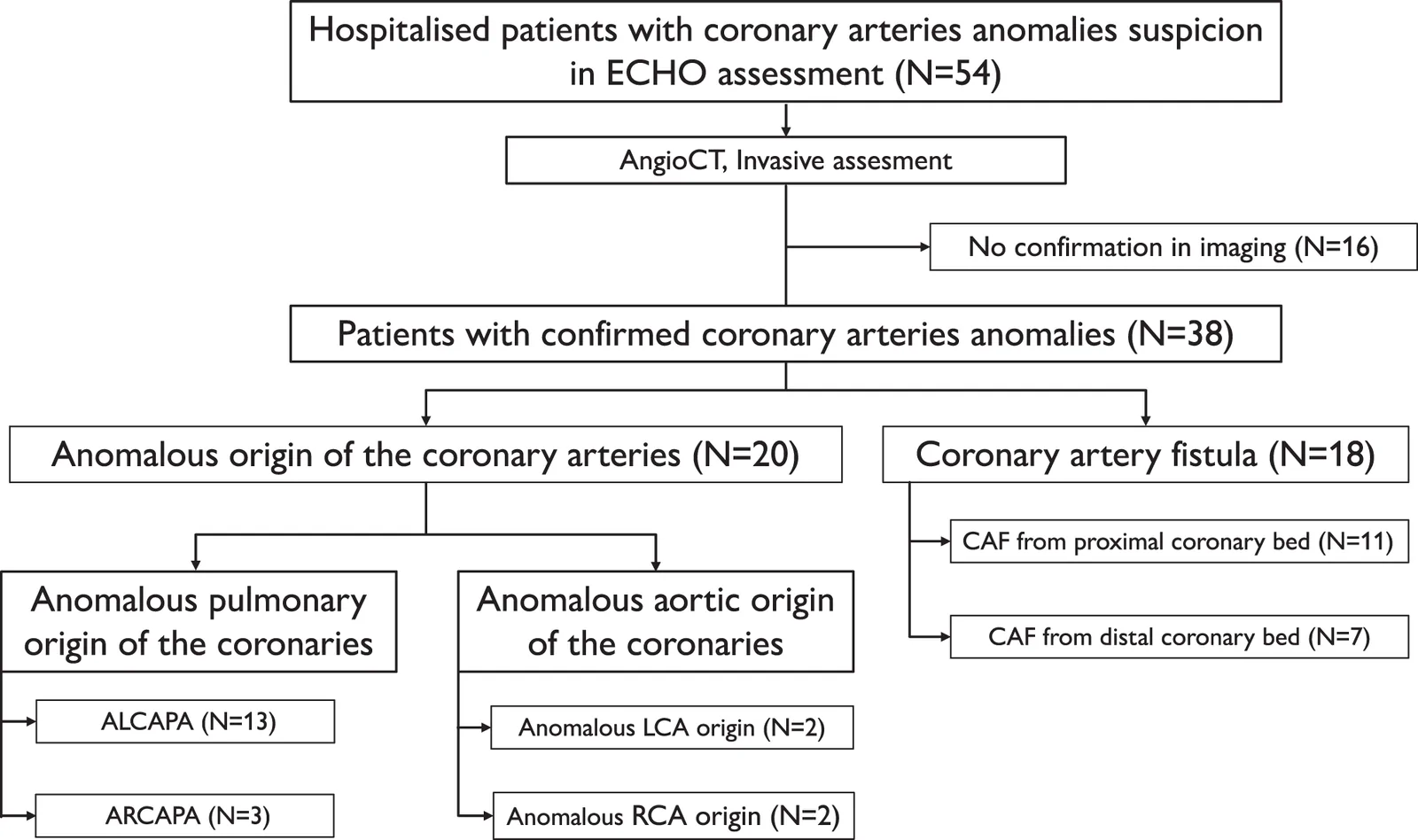
Flowchart with the enrolment. Abbreviations: ALCAPA, anomalous left coronary artery from the pulmonary Artery; ARCAPA, anomalous right coronary artery from the pulmonary Artery; angioCT, angiographic computed Tomography; CAF, coronary artery Fistula; ECHO, Echocardiography; LCA, left coronary Artery; RCA, right coronary artery

It was a single-centre, retrospective, cohort study that included 54 consecutive paediatric patients with a preliminary diagnosis of CAAs at the Department of Paediatric Cardiology, Poznan University of Medical Sciences, between 01.2012 and 03.2025. We included all patients admitted to the hospital due to a clinical presentation suggestive of CAAs and echocardiography (ECHO) assessment (Fig. 1). The inclusion criteria were: (I) age under 18 years; (II) CAAs diagnosis according to the International Classification of Diseases (ICD-10 code for primary diagnosis Q24.5). A confirmed case was defined as the presence of CAAs demonstrated on angioCT and/or cardiac catheterisation with coronary angiography. Exclusion criteria included lack of confirmation of CAAs on CT angiography or cardiac catheterisation with coronary angiography (16 of 54 patients were excluded; consequently, data of 38 patients were analysed). Patients meeting eligibility criteria were divided into groups using Gentile classification [5] and analysed in origin anomalies and CAF groups (no anomalies of course and coronary stenosis were found in our population).

The present research was conducted in accordance with the Declaration of Helsinki and was approved by the Ethics Committee of Poznan University of Medical Sciences (approval code 288/25).

### Clinical assessment

Detailed medical history was obtained by attending physicians upon admission, with a particular interest in identifying the specific type of CAAs. Blood pressure (BP), heart rate (HR), oxygen saturation (SpO_2_), height, and body weight were measured during the physical examination.

### Laboratory tests

Fasting blood samples were collected for the biochemical tests. The following laboratory parameters were of particular interest: haemoglobin, haematocrit, B-type natriuretic peptide (BNP) and high-sensitivity cardiac troponin I (hs-cTnI). Additionally, renal and liver function parameters and electrolytes (sodium, potassium) were assessed.

### Electrocardiography and echocardiography

Electrocardiography (ECG) and ECHO were performed in all of the enrolled patients several times during hospitalisation. In the ECG we assessed ischemic changes (pathologic Q wave, ST changes, T wave inversion), conduction changes (prolonged PQ interval, atrioventricular block, widened QRS) and hypertrophic changes (increased R wave amplitude in V6 and increased S wave depth in V1 considering the age of the patient, T wave inversion in V5 and V6 in case of the age of more than 2 days, deep Q wave more than 5 mm in II, III, aVF, V5, V6 or no Q waves in V5 and V6). Echocardiographic parameters which were collected are: mitral insufficiency (with stages), tricuspid insufficiency, dilatation of the left ventricle (by LV dimensions - left ventricle end diastolic diameter (LVEDd) and left ventricle end systolic diameter (LVESd) and their Z-scores), and ejection fraction assessed (Simpson formula). The left ventricular Z-scores were calculated using age‑ and sex‑specific reference values adjusted for body surface area, according to the regression model described by [6]. The threshold for left ventricular dilatation was defined as a Z‑score > +2. Mild regurgitation (stage I/II) was defined according to standard paediatric echocardiographic criteria, including a small color Doppler jet area and a narrow vena contracta. Moderate‑to‑severe regurgitation (stage III/IV) was defined by a larger jet area, increased vena contracta width, and supportive Doppler findings, such as holosystolic flow reversal when present.

### AngioCt/cardiac catheterisation

In all patients with suspected CAAs, diagnosis was confirmed using CT angiography or cardiac catheterisation with coronary angiography. The choice of method depended on the patient’s clinical condition and the availability of the test. The final decision was based on an individualized approach. In cases where the angioCT findings remained uncertain, four patients subsequently underwent cardiac catheterisation, which ultimately established the diagnosis.

### Medical history, hospitalisation and follow-up

The medical history was completed with the data from hospital databases by a research team to provide detailed information on hospitalisation. Data regarding the perioperative course were collected, including perioperative complications, extracorporeal membrane oxygenation (ECMO) use, and intensive care unit (ICU) stay in surgical patients. Furthermore, discharge treatment and length of hospitalisation were recorded for all patients.

The survival was verified in the Polish National Health Fund registry. Most patients were followed either through hospital readmission or outpatient visits, typically until 18 years of age. All patients, except for the three patients with ARCAPA who continued care at another centre, underwent at least one follow-up assessment one year after hospitalisation, most patients had at least two assessments. During follow‑up, they were evaluated for symptoms and neurological complications and underwent echocardiography with ejection fraction measurement. One patient was lost to follow‑up and assessed only for survival in the Polish National Health Fund registry, while three remained under the care of another centre.

### Statistical analysis

Statistical analyses were conducted using STATISTICA 13.3 (Tibco Software Inc., Palo Alto, CA, USA) and PQStat Software 2023 (v1.8.6; Poznan, Poland). Normal distribution was tested using the Shapiro-Wilk test to all continuous variables. Appropriate histograms are presented for the most important variables. The variables with normal distribution were presented via means ± standard deviation (SD) and compared with the independent samples Student’s t-test. The Mann–Whitney U-test was used to analyse non-normally distributed variables, and the results were expressed as median and interquartile range. Categorical variables are presented as numbers of cases and corresponding percentages in brackets. Pearson’s χ 2 tests or Fisher’s exact test were applied to compare categorical factors between groups. The number of subjects with missing data is presented in tables in square brackets for each group. The Levene tests were used to check the homogeneity of the variance.

Statistical significance was defined as *p* < 0.05.

## Results

The study cohort contained 38 consecutive, hospitalised paediatric patients: 42% males and 58% females. The mean age of the analysed group was 3.32 ± 5.35 years (min. 1 day, max. 16 years; IQR 0–4.25), 43.14 ± 64.14 (min. 1 day max. 196 months; IQR 1.75–11.75) months.

In our study, we recruited all patients with suspected CAAs based on echocardiographic assessment. We divided all of the patients into two subgroups, according to the results of invasive and non-invasive radiological imaging. We included 20 patients with origin anomalies and 18 patients with CAF. In the origin anomalies group, 16 patients had anomalous pulmonary origin of the coronary arteries: 13 patients had anomalous left coronary artery from the pulmonary artery (ALCAPA) and 3 patients had anomalous right coronary artery from the pulmonary artery (ARCAPA). The other 4 patients had origin anomalies. Two of them related to an anomalous left coronary artery (LCA) origin: one from the right aortic sinus of Valsalva and the second without a left coronary trunk, the remaining two related to an anomalous right coronary artery (RCA) origin: a single coronary artery arising from the right aortic sinus of Valsalva and the other of an ectopic RCA from the aorta. In the CAF group, we enrolled 18 patients: 11 patients with CAF from the proximal coronary bed and 7 patients from the distal coronary bed. The enrolment process is presented in Fig. 1. Baseline characteristics of the studied group are presented in Table 1. Diagnostic and treatment information of the studied group is presented in Table 2.

**Table 1:** Baseline characteristic of the studied group

**Characteristic**^**a**^	All (N = 38)	Anomalies of origin (N = 20)	Coronary artery fistulas (N = 18)	p
Age (years)	3.05 ± 5.26	2.60 ± 5.29	3.56 ± 5.33	0.223
- 0–12 months	57.90% (*N* = 22)	70% (*N* = 14)	44.44% (*N* = 8)	0.111
- 1–3 years	15.79% (*N* = 6)	10% (*N* = 2)	22.22% (*N* = 4)	0.558
- 4–6 years	7.90% (*N* = 3)	5% (*N* = 1)	11.11% (*N* = 2)	0.924
- 7–12 years	7.90% (*N* = 3)	5% (*N* = 1)	11.11% (*N* = 2)	0.924
- 13–18 years	10.53% (*N* = 4)	10% (*N* = 2)	11.11% (*N* = 2)	0.676
Age (months)	39.98 ± 62.79	33.85 ± 62.29	46.81 ± 64.44	0.383
Sex (male)	42.11% (*N* = 16)	40% (*N* = 8)	44.44% (*N* = 8)	0.959
HR on admission (bpm)	120.54 ± 37.85	125.89 ± 36.63	114.88 ± 39.42	0.398
SpO2 on admission (%)	99 (96–100)	99 (96–100)	100 (96.5–100)	0.617
Weight (kg)	6.2 (4.5–15)	6 (5.02–12.26)	7.1 (3.65–18.5)	0.941
**Symptoms at the admission**				
Dyspnoea	31.58% (*N* = 12)	50% (*N* = 10)	11.11% (*N* = 2)	**<0.001**
Cyanosis	13.16% (*N* = 5)	25% (*N* = 5)	0% (*N* = 0)	**0.048**
Syncope	2.63% (*N* = 1)	5% (*N* = 1)	0% (*N* = 0)	0.526
Chest pain	5.26% (*N* = 2)	0% (*N* = 0)	11.11% (*N* = 2)	0.218
No weight gain	10.53% (*N* = 4)	10% (*N* = 2)	11.11% (*N* = 2)	0.911
Feeding problems	10.53% (*N* = 4)	15% (*N* = 3)	5.56% (*N* = 1)	0.604
Fatigue	31.58% (*N* = 12)	45% (*N* = 9)	16.67% (*N* = 3)	0.074
Lack of symptoms	42.11% (*N* = 16)	35% (*N* = 7)	50% (*N* = 9)	0.512
**Comorbidities**				
Coexisting Congenital Heart Defect	13.16% (*N* = 5)	15% (*N* = 3)	11.11% (*N* = 2)	0.723
- PFO	7.90% (*N* = 3)	5% (*N* = 1)	11.11% (*N* = 2)	0.595
- PDA	2.63% (*N* = 1)	5% (*N* = 1)	0% (*N* = 0)	0.336
- ASD	5.26% (*N* = 2)	5% (*N* = 1)	5.56% (*N* = 1)	0.939
Infection	10.52% (*N* = 4)	20% (*N* = 4)	0% (*N* = 0)	0.107

Comparison of baseline characteristic parameters between two groups

a. Data presented as XX ± YY follow a normal distribution (XX—mean, YY—standard deviation), and data presented as XX (YY–ZZ) do not follow standard distribution (XX—median, YY—lower quartile, ZZ—higher quartile). The count of subjects for categorical variables are presented as (*N* = XX) below percentage. The count of subjects for each variable with data lacking is presented as a [number] in square brackets for each group. If data are present for the entire group, no square brackets are presented

Abbreviations: ASD, Atrial Septal Defect; AST, PDA, Patent Ductus Arteriosus; PFO, Patent Foramen Ovale; SpO2, Oxygen Saturation

**Table 2:** Diagnostic and treatment information of the studied group

**Characteristic**^**a**^	All (N = 38)	Anomalies of origin (N = 20)	Coronary artery fistulas (N = 18)	p
**ECG changes**				
Ischemic changes	36.84% (*N* = 12)	50% (*N* = 10)	22.22% (*N* = 4)	0.151
- pathologic Q wave	26.32% (*N* = 10)	50% (*N* = 10)	0% (*N* = 0)	**<0.001**
- ST changes	28.95% (*N* = 11)	45% (*N* = 9)	11.11% (*N* = 2)	**0.024**
- T wave inversion	7.90% (*N* = 3)	0% (*N* = 0)	16.67% (*N* = 3)	0.096
Conduction changes	10.53% (*N* = 4)	5% (*N* = 1)	16.67% (*N* = 3)	0.328
Hypertrophic changes	13.16% (*N* = 5)	15% (*N* = 3)	11.11% (*N* = 2)	0.723
Any deviations ^**b**^	52.63% (*N* = 20)	60% (*N* = 12)	44.44% (*N* = 8)	0.526
**Echocardiographic parameters**				
Mitral insufficiency	39.47% (*N* = 15)	65% (*N* = 13)	11.11% (*N* = 2)	**0.001**
- stage I/II	18.42% (*N* = 7)	20% (*N* = 4)	16.67% (*N* = 3)	0.566
- stage III/IV	26.32% (*N* = 10)	45% (*N* = 9)	5.55% (*N* = 1)	**0.006**
Tricuspid insufficiency	2.63% (*N* = 1)	5% (*N* = 1)	0% (*N* = 0)	0.336
Dilatation of the left ventricle	28.95% (*N* = 11)	55% (*N* = 11)	0% (*N* = 0)	**<0.001**
LVEDd	3.35 ± 0.91	3.48 ± 0.75	3.19 ± 1.08	0.347
LVEDd z-score	3.078 ± 4.652	5.044 ± 5.554	0.764 ± 1.337	**0.023**
LVESd	2.26 ± 0.78	2.56 ± 0.78	1.88 ± 0.62	0.008
LVESd z-score	4.192 ± 7.749	7.507 ± 9.062	0.049 ± 1.763	**0.004**
EF on admission	70 (56–73.75)	61.5 (43.5–69.75)	73 (71.25–75.75)	**<0.001**
EF lowest during hospitalisation (%)	66.5 (49.25–72.75)	52.5 (35–67.25)	70.5 (66.25–73)	**0.001**
EF highest during hospitalisation (%)	72 (67.75–75.25)	72 (64–72.5)	74 (70–81)	**0.015**
EF improvement (%)	7.5 (0–13.5)	10.5 (2.25–29.5)	0.5 (0–8)	**0.032**
EF < 50%	26.32% (*N* = 10)	45% (*N* = 9)	5.56% (*N* = 1)	**0.009**
**Comorbidities**				
Coexisting Congenital Heart Defect	13.16% (*N* = 5)	15% (*N* = 3)	11.11% (*N* = 2)	0.723
PFO	7.90% (*N* = 3)	5% (*N* = 1)	11.11% (*N* = 2)	0.595
PDA	2.63% (*N* = 1)	5% (*N* = 1)	0% (*N* = 0)	0.336
ASD	5.26% (*N* = 2)	5% (*N* = 1)	5.56% (*N* = 1)	0.939
Infection	10.52% (*N* = 4)	20% (*N* = 4)	0% (*N* = 0)	0.107
**Diagnosis**				
AngioCT	53.16% (*N* = 20)	60% (*N* = 12)	44.44% (*N* = 8)	0.526
Cardiac Catheterisation with coronary angiography	47.37% (*N* = 18)	40% (*N* = 8)	55.55% (*N* = 10)	0.526
Time from suspicion to diagnosis (days)	3 (2–6)	2.5 (2–4.75)	4 (2–6)	0.379
**Treatment**				
Conservative treatment	42.11% (*N* = 16)	25% (*N* = 5)	61.11% (*N* = 11)	**0.027**
Invasive intervention	57.89% (*N* = 22)	75% (*N* = 15)	38.89% (*N* = 7)	**0.027**
- Surgical correction^c^	52.63% (*N* = 20)	75% (*N* = 15)	27.78% (*N* = 5)	**0.008**
- Transcatheter intervention^c^	7.90% (*N* = 3)	0% (*N* = 0)	16.67% (*N* = 3)	0.097
Length of hospitalisation ^d^ (days)	9.4 (2.20–27.50) [3]	27 (6–38.55) [3]	5.2 (2.04–11.81)	**0.008**
Death ^e^	0	0	0	
**Medications after discharge**				
Loop diuretics	15.79% (*N* = 6)	25% (*N* = 5)	5.56% (*N* = 1)	0.184
β-Blocker	5.26% (*N* = 2)	10% (*N* = 2)	0% (*N* = 0)	0.488
MRA	39.47% (*N* = 15)	45% (*N* = 9)	33.33% (*N* = 6)	0.687
ACEI	28.95% (*N* = 11)	45% (*N* = 9)	11.11% (*N* = 2)	**0.032**
Digoxin	5.26% (*N* = 2)	10% (*N* = 2)	0% (*N* = 0)	0.488
ASA	21.05% (*N* = 8)	15% (*N* = 3)	27.78% (*N* = 5)	0.571
Anticoagulants^f^	23.68% (*N* = 9)	30% (*N* = 6)	16.67% (*N* = 3)	0.454
**Biochemical parameters**				
BNP on admission (pg/mL)	66.03 (25.4–410.6) [12]	175 [29.7–814.1] [5]	36 [27.2–79.4] [7]	0.138
BNP highest during hospitalisation (pg/mL)	121.3 (23.6–1488.25) [12]	635.2 [29.7–4244.6] [5]	35 [24.4–198.25] [7]	0.212
Troponin on admission (μg/L)	53.6 (9.68–272.18) [12]	197 [46.9–594.5] [5]	16.4 [6.7–57.2] [7]	0.601
Troponin highest during hospitalisation (μg/L)	84.5 (10.65–1465.15) [12]	979 [187.63–8417] [5]	50 [6.7–84.5] [7]	0.217
HCT (L/L)	35.29 ± 5.12	33.26 ± 5.04	38.25 ± 3.68	**0.006**
Hgb (mmol/L)	12.32 ± 1.83	11.61 ± 1.89	13.36 ± 1.15	**0.005**

Comparison of diagnostic and treatment characteristic parameters between two groups

a Data presented as XX ± YY follow a normal distribution (XX—mean, YY—standard deviation), and data presented as XX (YY–ZZ) do not follow standard distribution (XX—median, YY—lower quartile, ZZ—higher quartile). The count of subjects for categorical variables are presented as (*N* = XX) below percentage. The count of subjects for each variable with data lacking is presented as a [number] in square brackets for each group. If data are present for the entire group, no square brackets are presented

b “at least one of: ischemic, conduction, or hypertrophic changes”

c Analysis performed only among patients with performed intervention. One patient underwent both transcatheter and surgical intervention

d ARCAPA patients were excluded from the perioperative and postoperative analysis due to transport to other surgical center

e No death in any of our patients were noted in the data obtained from the Polish National Health Fund registry

f Warfarin

Abbreviations: ACEI, Angiotensin-Converting Enzyme Inhibitor; ASA, Acetylsalicylic Acid; BNP, B-type Natriuretic Peptide; ECG, Electrocardiogram; ECMO, Extracorporeal Membrane Oxygenation; EF, Ejection Fraction; HCT, Haematocrit; Hgb, Haemoglobin; HR – Heart Rate; ICU, Intensive Care Unit; LVEDd, left ventricle end diastolic diameter; LVESd, left ventricle end systolic diameter; MRA, Mineralocorticoid Receptor Antagonist

**Fig. 2:**
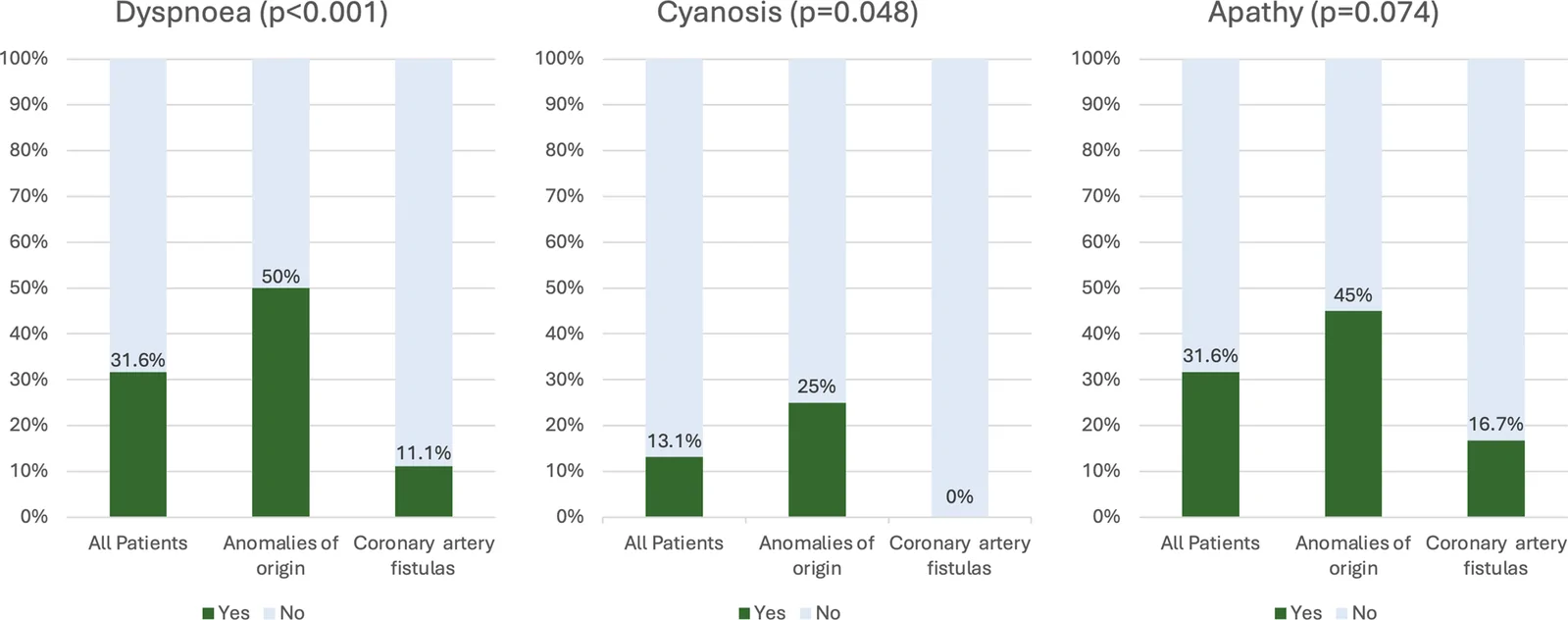
Comparison of selected symptoms presented on admission depending on diagnosis in the whole group of patients (*n* = 38)

42.1% of the patients were asymptomatic on admission and their hospitalisation was based on imaging findings. In fact, half of the CAF patients were asymptomatic, while only 35% of origin anomalies patients did not present any symptoms (*p* = 0.512). All asymptomatic patients were referred to the clinic due to cardiac murmurs. Among symptomatic patients, the most commonly reported symptom was dyspnoea, which was present in 31.6% of participants. Patients with origin anomalies reported this symptom more frequently than the CAF group (*p* < 0.001). Cyanosis was also more common in the origin anomalies group (*p* = 0.048). 31.6% of patients presented with fatigue, 45% in the origin anomalies group and 16.7% in the CAF group (*p* = 0.074). Comparison of those parameters is presented in Fig. 2. Other symptoms reported by the patients were feeding problems (10.5%), failure to gain weight (10.53%), chest pain (5.3%) and syncope (2.6%). Syncope was reported only by the origin anomalies patients (*p* = 0.526), whereas chest pain was presented only in the CAF group (*p* = 0.218).

Coexisting congenital heart defects (CHD) were observed in 13.2% of patients, 15% vs 11.1% in the subgroups (*p* = 0.723). The most common were patent foramen ovale (7.9%), atrial septal defect (5.3%) and patent ductus arteriosus (2.6%). None of our patients were diagnosed with tetralogy of Fallot, transposition of the great arteries or any other complex heart defect. There was no significant difference between the two groups in any of the defects. In addition, active infection was present in 10.5% on admission, with a predominance in the origin anomalies group (20% vs 0%, *p* = 0.107).

The ECG examination showed significant changes in 20 patients (52.6%): 14 patients (36.8%) had ischaemic changes, 4 patients (10.5%) had conduction changes, and 5 patients (13.2%) had hypertrophic changes. Pathologic Q waves (50% vs. 0%, *p* < 0.001) and ST segment changes (45% vs. 11.11%, *p* = 0.024) occurred more frequently in the origin anomalies group. T wave inversion was present more often in the CAF group (0% vs 16.7%, *p* = 0.096), however it was statistically insignificant.

There were significant differences in echocardiographic assessment between the two groups. Mitral regurgitation was more frequent in patients with origin anomalies (65% vs 11.1%; *p* = 0.001), as well as ventricular dilatation (55% vs 0%; *p* < 0.001). LVEDd and LVESd Z-scores were greater in children with origin anomalies compared with the CAF group, and these differences reached statistical significance (LVEDd Z-score 5.044 vs 0.764, *p* = 0.023 and LVESd Z-score 7.507 vs 0.049, *p* = 0.004). However, only severe mitral regurgitation (stage III/IV) was more often presented in origin anomalies patients (45% vs 5.55%, *p* = 0.006), where milder stages (stage I/II) were presented without significant differences in both groups (*p* = 0.566). Tricuspid regurgitation (stage II) was also present in one patient with origin anomalies (*p* = 0.336). In addition, patients with origin anomalies had a lower ejection fraction (EF) on admission, with a median EF of 61.5% compared to patients with CAF, where the median EF was 73% (*p* < 0.001). The minimal in-hospital EF was also lower in the origin anomalies group (median 52.5% vs. 70.5%; *p* = 0.001). Only 45% of patients with origin anomalies had a reduced EF, probably due to the short time from diagnosis to surgery, which prevented them from developing AHF. The average waiting time of patients with ALCAPA for surgery from diagnosis was 5 days, while 6 patients underwent surgery within 48 hours.

The final diagnosis was made after coronary computed tomography angiography in 52.6% of patients and by cardiac catheterisation with coronary angiography in 47.4% of patients. The median time from the initial suspicion of the disease to the final diagnosis was 3 days and did not differ between groups (*p* = 0.379).

**Fig. 3:**
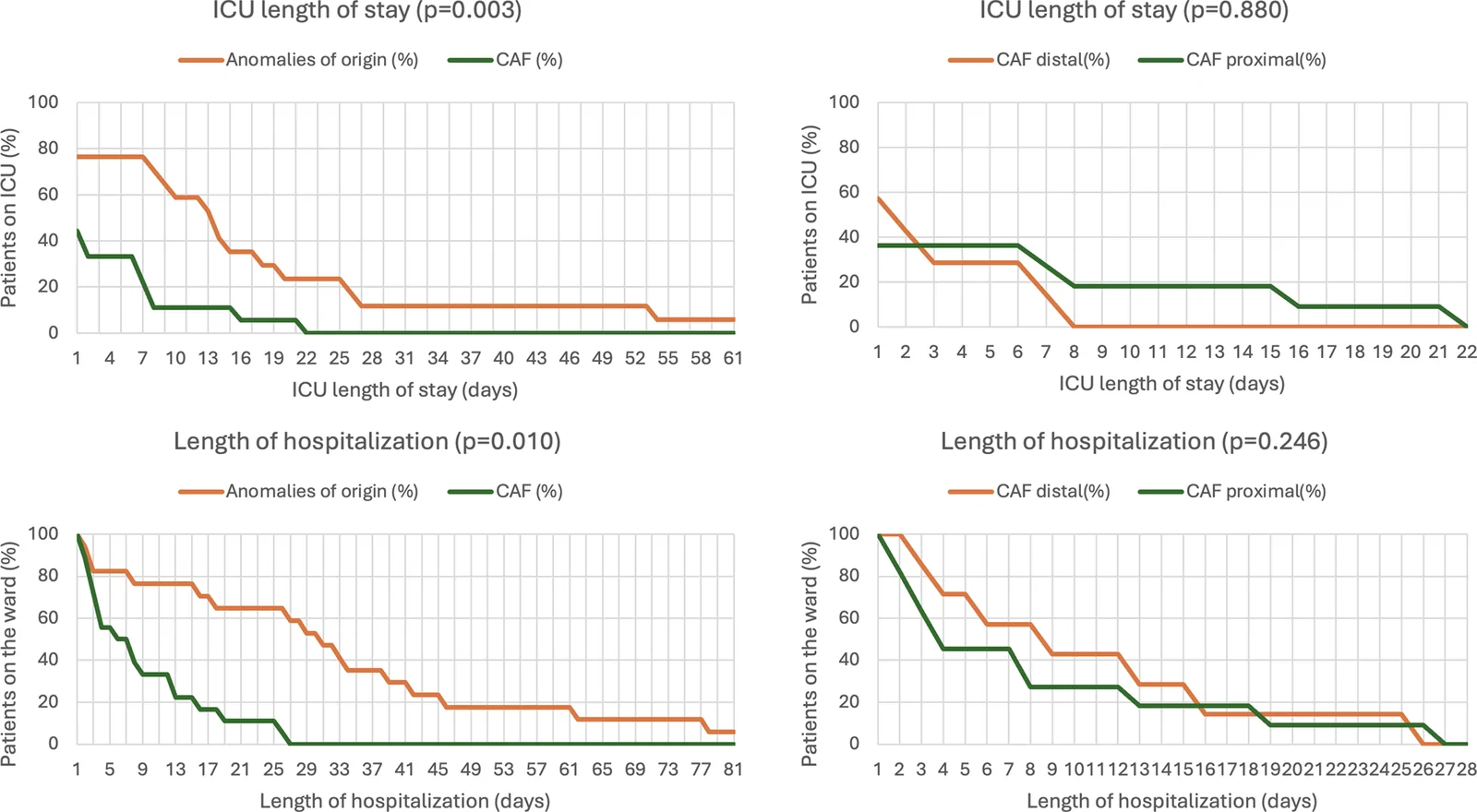
Comparison of length of stay in the ICU and hospitalisation between different diagnoses. *Patients with ARCAPA were excluded from this analysis due to transport of the patients to the other cardiac surgery centre. Abbreviation: ICU – Intensive Care Unit

**Fig. 4:**
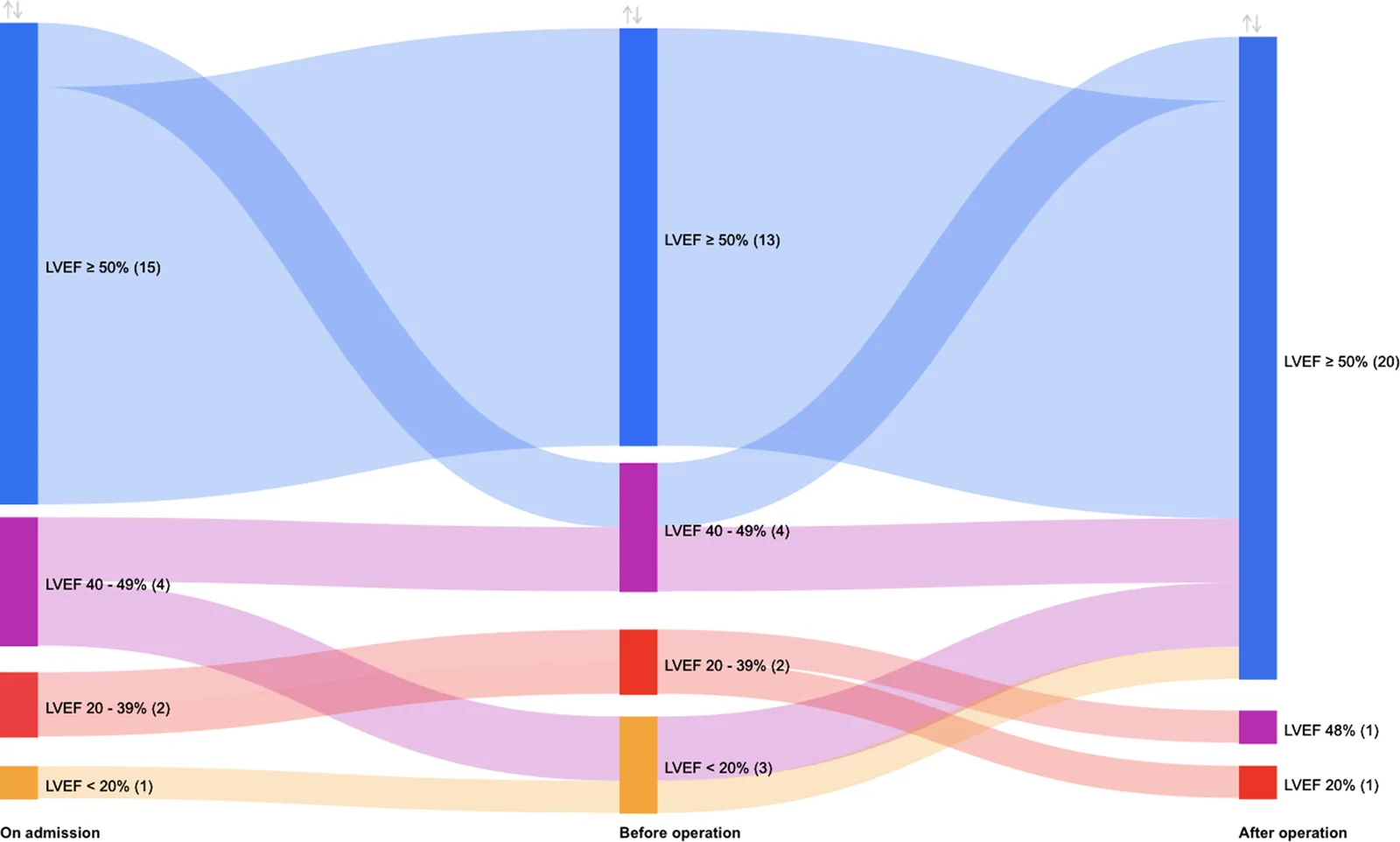
Sankey chart - LVEF change on admission, before operation and after operation among operated patients. *Number of patients to each group in the bracket. Abbreviation: LVEF - left ventricle ejection fraction

57.9% of the studied group were treated with invasive intervention (*N* = 22), whereas 42.1% (*n* = 16) were observed without any intervention. Invasive interventions were more commonly performed in the origin anomalies group (75% vs 38.8%, *p* = 0.047). In turn, conservative treatment was more frequent in CAF patients (11 CAF patients vs 5 origin anomalies patients). Origin anomalies were more frequently treated with surgical intervention than CAF (75% vs 27.8%, *p* = 0.080). On the other hand, 16.7% CAF were treated with the transcatheter intervention. One patient in the CAF group required both interventional and surgical treatment. Four patients required re-intervention after primary correction: 3 were diagnosed with origin anomalies and 1 with CAF. All reinterventions were made by surgical intervention. 84.61% of patients with origin anomalies required ventilatory support during hospitalisation, in contrast to CAF patients who required it in 57.14% of cases (*p* = 0.337). In addition, 92.3% of the origin anomalies group required inotropic support, compared to none of the CAF group (*p* < 0.001). Among the operated patients, most of the origin anomalies group required perioperative VA-ECMO (61.54% vs 0%, *p* = 0.015). Perioperative complications that occurred in the surgical patients included AHF (4 patients), severe arrhythmias (2 patients), and low cardiac output syndrome (5 patients). 15 patients required respiratory support, and 8 patients required ECMO. Patients who required ECMO had more perioperative complications than other surgical patients. Brief characteristics of the patients requiring ECMO are presented in Table 3. All operated patients required admission to the ICU. Patients with an origin anomalies had a significantly longer ICU stay than patients with CAF (median 14 days vs 7 day, *p* = 0.026), as well as the hospitalisation length (median 27 days vs 5.2 days, *p* = 0.008). Patients with ARCAPA were excluded from the analysis of perioperative courses due to transfer to another cardiac surgery centre and lack of relevant data; however, we analysed the long-term follow-up of these patients. Detailed analysis is shown in Figure 3 and Table 4. Left ventricle EF changes on admission, before operation and after operation among operated patients are presented in Fig. 4. Patients with origin anomalies who did not require surgical intervention presented with mild defects that were not associated with significant clinical symptoms. Almost all of these patients were stable and did not require medication. Only one patient had an ejection fraction of 42% and lisinopril was started; his condition was assessed as good. One of these patients has been lost to follow-up, but The National Health Fund registry indicates that he is alive.

**Table 3:** Characteristics of patients undergoing extracorporeal membrane oxygenation (ECMO)

Characteristic	All (*N* = 8)
Indications	
- Low cardiac output syndrome (LCOS)	62.5% (*N* = 5)
- Acute left heart failure	25% (*N* = 2)
- Ventricular Arrhythmias	12.5% (*N* = 1)
Group of patients - Anomalies of origin - ALCAPA	100% (*N* = 8)
Timing of initiation - Immediately upon completion of the surgical procedure	100% (*N* = 8)
Days of treatment (days)	5 (min. 4 - max. 8)

**Table 4:** Perioperative outcomes and postoperative complications in patients treated invasively in our centre

Characteristic *	All (N = 20)	Anomalies of origin (N = 13)	Coronary artery fistulas (N = 7)	p
Perioperative ECMO	40% (*N* = 8)	61.54% (*N* = 8)	0% (*N* = 0)	**0.015**
Ventilatory support	75% (*N* = 15)	84.61% (*N* = 11)	57.14% (*N* = 4)	0.337
Inotropic agents	60% (*N* = 12)	92.3% (*N* = 12)	0% (*N* = 0)	**<0.001**
ICU length of stay (days)	13 (7–19.5)	14 (12–25)	7 (6–11)	**0.026**
Postoperative complications	45% (*N* = 9)	61.15% (*N* = 8)	14.29% (*N* = 1)	0.070
− Neurological deficits	25% (*N* = 5)	38.46% (*N* = 5)	0% (*N* = 0)	0.083
− Thrombosis	15% (*N* = 3)	15.38% (*N* = 2)	14.29% (*N* = 1)	1.000
− Venous Thrombosis	10% (*N* = 2)	15.38% (*N* = 2)	0% (*N* = 0)	0.416
− Arterial Thrombosis	5% (*N* = 1)	0% (*N* = 0)	14.29% (*N* = 1)	0.350
− Mediastinitis	5% (*N* = 1)	7.69% (*N* = 1)	0% (*N* = 0)	0.650
− Reinterventions	20% (*N* = 4)	23.07% (*N* = 3)	14.29% (*N* = 1)	0.614

*Analysis performed only among patients with performed intervention in our centre. ARCAPA patients were excluded from the perioperative and postoperative analysis due to transport to other surgical centre (2 patients with ARCAPA were operated in other centre, one patient with ARCAPA was lost to follow-up but The National Health Fund registry indicated that he is alive)

Among patients who required interventional treatment, 45% of the patients (*N* = 9) presented postoperative complications (*p* = 0.070). 25% patients (*N* = 5) presented new neurological symptoms postoperatively. All of them were in the origin anomalies group (*p* = 0.083). Other postoperative complications include thrombosis (15%) and mediastinitis (5%) with no differences among groups. Venous thrombosis appeared only in origin anomalies patients (*p* = 0.416), whereas arterial thrombosis appeared only in CAF patients (*p* = 0.350). (Details are shown in Table 4.)

Medications prescribed on discharge were mostly similar in both groups. Only angiotensin-converting enzyme inhibitors (ACEI) were used more often in the origin anomalies group than in the CAF group (45% vs 11.1% respectively; *p* = 0.032). No angiotensin II receptor blockers (ARB) were used in our study.

Moreover, markers of cardiac injury, BNP and troponin levels, were similar in both groups, not only on admission, but also during hospitalisation. The only difference in the biochemical parameters measured was in the morphological parameters. Haematocrit levels on admission were lower in the origin anomalies group (*p* = 0.006), as was haemoglobin on admission (*p* = 0.005). It should be noted that the comparison between origin anomalies and CAF remains exploratory and may be confounded by disease severity and age distribution.

All enrolled patients were alive as of March 2025 at the end of the observational study according to the Polish National Health Fund registry.

## Discussion

Our study emphasises that the early diagnosis of some CAAs, including ALCAPA, was associated with clinical improvement and no mortality observed during the follow-up period in the study group. To the best of our knowledge, this is among the first studies to compare the origin anomalies with anomalies of termination like CAF. We also provide an accurate analysis of the perioperative and postoperative period of patients who required surgical treatment. Despite the huge heterogeneity of the anomalies of the coronary arteries, we used the simplified classification proposed by [5]. Although our cohort didn’t have anomalies of the coronary artery course and coronary stenosis, we compared anomalies of artery origin with anomalies of artery termination, represented in our work by coronary arteriovenous fistulas. Implemented comparison enables us to provide novel findings beyond the standard descriptions provided in the literature. To the best of our knowledge, this is among the first studies that shows that haemoglobin levels and haematocrit were statistically significantly lower in origin anomalies than in CAF. Moreover, patients with origin anomalies had statistically more frequent dyspnoea and cyanosis, as well as mitral insufficiency, lower EF and dilatation of left ventricle. Importantly, there were no differences between natriuretic peptides.

In the literature, there are few studies describing CAAs in children, however, they differ from our work. Moreover, most of these studies were performed in the distant past and comprised slightly different groups of patients. Our study population consisted of 20 patients with origin anomalies and 18 patients with CAF. In our study, 15 patients with origin anomalies, including 13 patients with ALCAPA, 2 ARCAPA and 5 patients with CAF, underwent surgical treatment. Consecutively, 3 patients with CAF underwent transcatheter intervention. One patient underwent both transcatheter and surgical intervention. All of them were operated on with good results and no one died. Nevertheless, we must point out that some of our patients developed postoperative complications, including neurological deficits (25%), thrombosis (15%), mediastinitis (5%) and some patients required reinterventions (20%).

In the study of Werner et al., 29 patients were diagnosed with abnormalities of the coronary arteries between 1988 and 1999 [7]. The studied population consisted of 9 patients with CHD and coexisting abnormalities of coronary arteries, 2 patients with Kawasaki Disease and 18 children with isolated CAAs, including 13 patients with ALCAPA, 1 patient with ARCAPA and 4 patients with CAF. In contrast to our work where only patients with congenital abnormalities were included. Moreover, their study demonstrates a significantly higher postoperative mortality rate, which reflects the substantial advancements in surgical techniques and perioperative care in recent years [7].

In turn, in the study of Uysal et al. the population consists of 22 patients diagnosed between 1993 and 2013. However, they excluded patients with CHD [8]. Furthermore, they have a significantly higher mortality rate (18.1%), one patient died during the preoperative period and 2 of 7 patients with ALCAPA died during the postoperative period [8]. However, they did not show more data about the time from diagnosis to treatment and the postoperative period including complications [8].

Perioperative complications following operations of ALCAPA and other anomalies of the coronary arteries, are not commonly described in the literature. Nevertheless, there are some older reports, like De Wolf et al. with an accurate description of postoperative follow-up. De Wolf et al. presented a population consisting of 33 patients, including 15 with ALCAPA, 12 with CAF and 6 with coronary stenosis and/or thrombosis [9]. The study was conducted between 1990 and 2001. 14 patients with ALCAPA were operated on, one patient did not survive the operation, 2 patients had mild HF lasting beyond 6 months and 6 patients had mild mitral incompetence [9]. Moreover, in their study, one child with coronary stenosis died after heart catheterisation due to myocardial infarction. In contrast, their study included only symptomatic patients between 1 day and 14 years, they excluded patients with CHD. In contrast, in our work we included patients with CHD and also asymptomatic [9]. In fact, other reports suggest that the occurrence of the CAAs is not associated with other CHD [5, 10].

In our study, we decided to present the postoperative complications experienced by our patients who underwent surgery for CAAs. However, we did not observe any significant differences between the groups. Patients in the origin anomalies group were more likely to present with neurological deficits and mediastinitis, and to require reinterventions. There are two possible reasons for this. Firstly, origin anomalies patients were more likely to undergo surgical correction (only one patient received endovascular treatment). Secondly, origin anomalies patients, especially those with ALCAPA or ARCAPA, presented with preoperative HF in 45% of cases, with an impaired ejection fraction. They were mostly admitted to the hospital with signs of hemodynamic instability and management was urgent. It is not without reason that this type of CAAs is classified by Feng et al. as serious and potentially life-threatening [3]. The findings of Feng are coherent with our observations. Patients with origin anomalies spend more time in the hospital and in the ICU than patients with CAF. Nevertheless, the length of stay in the ICU among paediatric patients was significantly shorter compared to other studies, which may be attributed to the improvement of care [11, 12]. In our study, we analysed both children who underwent surgery and those who did not. To avoid generalization, we showed a detailed analysis in Fig. 3.

In our study, the prognosis of patients was better than in the previous works. It is worth noting that none of the patients in our study died. It may have emphasised the utility of early diagnosis and rapid treatment which was practised in our department. Moreover, good outcomes of surgical treatment may be a result of progress in cardiosurgical methods of operation including the use of ECMO and improvement of the quality of perioperative care nowadays.

It could also be a result of strict cooperation between paediatric cardiology and paediatric cardiac surgery in our hospital. As a standard practice in our centre, regular cardiology consultations following cardiac surgery have been established, with clinical decisions made jointly through interdisciplinary collaboration.

Our study showed that dyspnoea and cyanosis were statistically more common in patients with origin anomalies than in those with CAF. However, chest pain was observed only in patients with CAF, especially in those older. In turn, younger patients with HF, who constituted most of the origin anomalies population, presented with dyspnoea, feeding problems, and fatigue. Differences in symptom presentation may reflect the age difference between the groups and the coexistence of age‑specific symptoms, which could confound the comparison between groups. Furthermore, our study showed that 42.1% of patients with CAAs, including 35% of patients with origin anomalies, and 50% of patients with CAF were asymptomatic, which underscores the need for vigilance. Fortunately, the incidence of CAAs among children is low, as Davis emphasizes in his study [13]. They examined only asymptomatic children in the general population, in which only 4 patients (0.17%) were diagnosed with CAAs [13].

In turn, Temel et al. analysed data from 515 patients with CHD including mostly tetralogy of Fallot who underwent angiography and catheterisation between 2005 and 2009 [14]. 42 patients were diagnosed with coronary abnormalities including, 38 origin anomalies, 2 anomalies of intrinsic coronary arterial anatomy, and 2 anomalies of coronary termination. However, their origin anomalies differed from ours, there were no severe abnormalities including ALCAPA or ARCAPA. Instead, there were mainly patients with mild CAAs: mostly anomalous origin of both right and left coronary artery from aorta [14]. This situation does not lead to disruption of coronary circulation, consequently, does not cause the development of myocardial ischaemia or HF.

The pressing topic we have decided to address is also a comparison of the CAF distal vs. the CAF proximal. Gowda et al. showed that children with distal CAF are at higher risk of suboptimal remodelling after closure [15]. Li et al. stated that those CAF are at greater risk of thrombosis after closure [16]. In our study, 6 children were operated on surgically and 2 by transcatheter intervention. Moreover, thrombosis was the only postoperative complication more common in the CAF group and it occurred in the patient with distal type. Nevertheless, independently of the closure technique there is no difference between these two anatomical types of the CAF in length of hospitalisation and length of ICU stay. This may result from the limited size of the study population and require further investigation.

Furthermore, our study showed that patients with origin anomalies had significantly lower haemoglobin and haematocrit levels on admission than patients with CAF. In the literature, there is no work on the correlation between anaemia and CAAs. Kim et al. showed that patients with Kawasaki Disease with anaemia had a higher risk of coronary abnormalities [17]. However, the mechanism is suggested to be related to the immune system, which probably has no influence on our group of patients. Consequently, the mechanism of anaemia in origin anomalies is unknown. We hypothesise that it may be related to pre- and perioperative heart failure, potentially leading to impaired intestinal circulation with reduced nutrient absorption, renal ischemia, and generalised inflammation [18]. In support of this hypothesis, more origin anomalies patients in our cohort had EF < 50%, demonstrating circulatory failure. As we know, anaemia could be an adverse prognostic factor in HF with reduced ejection fraction [19]. In addition, anaemia may exacerbate myocardial ischemia caused by CAAs and consequently exacerbate clinical symptoms including dyspnoea. Nevertheless, these potential mechanisms remain speculative and require confirmation in future studies.

In turn, mitral regurgitation, reduced EF and left ventricle dilatation are typical consequences of origin anomalies, especially ALCAPA. Systolic dysfunction and left ventricle dilatation are consequences of myocardial ischemia and hypoperfusion, due to inappropriate coronary circulation. Mitral regurgitation is a result of dilatation of the annulus fibrosus or dysfunction of the papillary muscle. This has been shown in other studies [12, 20]. Nevertheless, these echocardiographic changes are very rare in CAF due to the lower impact of these anomalies on myocardial ischemia. Surprisingly, there was no significant difference in natriuretic peptides. This may be due to early diagnosis and prompt treatment, which do not lead to chronic HF.

## Limitations

Several limitations to this study should be noted. First, it was a single-centre analysis, which introduces the potential for selection bias. The second limitation is a relatively small number of patients; therefore, the results should be interpreted with caution and require confirmation in a larger multicentre cohort. Comparisons between subgroups are secondary and should be viewed as exploratory given the small cohort size. The main contribution of our study is the comprehensive description of patient characteristics and outcomes.

The third limitation is that ARCAPA treatment results were not included in our study due to transfer of patients to different centres and lack of perioperative data. However long-term follow-up of these patients is included in results. One patient with ARCAPA qualified for surgical treatment has been lost to follow-up, however The National Health Fund registry indicates that he is alive. The next limitation is that all detailed functional data from advanced Echo modalities were not included in our study. The next limitation is that no patients had major CHD such as tetralogy of Fallot (TOF) or dextro-transposition of the great arteries (DTGA) in our study. It remains uncertain whether the lack of cases was due to underdiagnosis of this patient group at our centre or to the methodology applied. Retrospective analysis of patients with DTGA and TOF showed that none of these patients had CAAs. This may reflect either a true absence of these anomalies or limitations in screening sensitivity or case detection.

We are aware that the applied comparison is simplified and represents both a strength and a weakness of our work. Considering these limitations, multicentre studies with a larger group of patients are required to validate the clinical value of our findings further.

## Conclusion

Our study demonstrates that timely, accurate diagnosis with modern imaging methods and immediate implementation of transcatheter or surgical treatment in patients who require it lead to favourable clinical recovery and no mortality observed during the follow-up period in the study group. We would like to emphasize that symptoms such as dyspnoea, cyanosis, syncope, no weight gain, feeding problems and fatigue, especially in younger children, should prompt us to expand the diagnosis and look for diseases such as CAAs. In addition, our results show a significant improvement in prognosis in patients who underwent surgical treatment. This may be related to the progress and improvement of surgical treatment methods, as well as improved perioperative care.

## Data Availability

The data that support the findings of this study are not openly available due to reasons of sensitivity and are available from the corresponding author upon reasonable request.

## Abbreviations

ALCAPA: Anomalous left coronary artery from pulmonary artery,
ARCAPA: Anomalous right coronary artery from the pulmonary artery,
BNP: B-type natriuretic peptide,
BP: Blood pressure,
CAAs: Coronary artery anomalies,
CAF: Coronary artery fistulae,
CHD: Congenital heart defects,
ECG: Electrocardiography,
ECHO: Echocardiography,
ECMO: Extracorporeal membrane oxygenation,
EF: Ejection fraction,
HF: Heart failure,
HR: Heart rate,
hs-cTnI: High-sensitivity cardiac troponin I,
ICU: Intensive care unit,
LCA: Left coronary artery,
LV: Left ventricle,
LVEDd: Left ventricle end diastolic diameter,
LVESd: Left ventricle end systolic diameter,
Origin anomalies: Anomalous origin of the coronary arteries,
RCA: Right coronary artery,
SD: Standard deviation,
SpO2: Oxygen saturation.
